# Surgical Management of Urolithiasis in Patients after Urinary Diversion

**DOI:** 10.1371/journal.pone.0111371

**Published:** 2014-10-31

**Authors:** Wen Zhong, Bicheng Yang, Fang He, Liang Wang, Sunil Swami, Guohua Zeng

**Affiliations:** 1 Department of Urology, the First Affiliated Hospital of Guangzhou Medical University, Guangdong Key Laboratory of Urology, Guangzhou, China; 2 Department of Gynecology and Obstetrics, the Third Affiliated Hospital of Guangzhou Medical University, Guangzhou, China; 3 Department of Biostatistics and Epidemiology, College of Public Health, East Tennessee State University, Johnson, Tennessee, United States of America; 4 Department of Epidemiology, College of Public Health and Health Professions, University of Florida, Gainesville, Florida, United States of America; Sun Yat-sen University, China

## Abstract

**Objective:**

To present our experience in surgical management of urolithiasis in patients after urinary diversion.

**Patients and Methods:**

Twenty patients with urolithiasis after urinary diversion received intervention. Percutaneous nephrolithotomy, percutaneous based antegrade ureteroscopy with semi-rigid or flexible ureteroscope, transurethral reservoir lithotripsy, percutaneous pouch lithotripsy and open operation were performed in 8, 3, 2, 6, and 1 patients, respectively. The operative finding and complications were retrospectively collected and analyzed.

**Results:**

The mean stone size was 4.5±3.1 (range 1.5–11.2) cm. The mean operation time was 82.0±11.5 (range 55–120) min. Eighteen patients were rendered stone free with a clearance of 90%. Complications occurred in 3 patients (15%). Two patients (10%) had postoperative fever greater than 38.5°C, and one patient (5%) suffered urine extravasations from percutaneous tract.

**Conclusions:**

The percutaneous based procedures, including percutaneous nephrolithotomy, antegrade ureteroscopy with semi-rigid ureteroscope or flexible ureteroscope from percutaneous tract, and percutaneous pouch lithotripsy, provides a direct and safe access to the target stones in patients after urinary diversion, and with high stone free rate and minor complications. The surgical management of urolithiasis in patients after urinary diversion requires comprehensive evaluation and individualized consideration depending upon the urinary diversion type, stone location, stone burden, available resource and surgeon experience.

## Introduction

Radical cystectomy has been a well established treatment option for invasive bladder cancer in clinical practice [1]. Postoperative changes in anatomy as well as other metabolic factors often result in urinary tract infection and urolithiasis in patients after urinary diversion [2]. The reported incidence of stones associated with urinary diversion ranges from 9% to 11% after ileal conduit diversion [3]–[4], 17% to 27% for pouch stones after Kock pouch diversion [5]–[6] and 11% to 12.9% after Indiana pouch diversion [6]–[7]. These stones also have a 33% to 63% recurrence rate within 3–5 years after the initial intervention [8]–[9].

The surgical management of stones in patients after urinary diversion is challenging. Open operation monotherapy has a limited role in the treatment of urolithiasis in these patients on account of the high recurrence rate of stones, postoperative scar, tissue adhesion and the changed anatomy. The advancement in equipments and increasing experience are making minimally invasive endourologic techniques an appropriate alternative choice for these cases as seen in several reports [9]–[12]. Recently, the introduction of flexible ureteroscopy, and new generation lithotripters including Ho: YAG laser and ultrasonic lithotripter of Swiss LithoClast Master have made the application of endourologic techniques in urinary tract stones treatment much more effective [13]–[14]. However, these techniques have not been well tested in patients with urolithiasis after urinary diversion.

In the present study, we present our experience in the management of urinary tract stones in patients after urinary diversion.

## Patients and Methods

Between January 2005 and December 2013, 20 patients with urinary tract stones after urinary diversion received intervention in the Department of Urology. Complete data was collected retrospectively, written informed consents were obtained from all participants, and the present study was approved by the Ethics Committee of the First Affiliated Hospital of Guangzhou Medical University.

The preoperative assessment included medical history, physical examination, complete blood count, urinary analysis, midstream urine culture and sensitivity test, coagulation profiles, electrolyte biochemical tests, ultrasonogrphy, abdominal plain X-ray film of kidney, ureter, and bladder (KUB). Intravenous urography (IVU) was required if serum creatinine (SCr) was normal. All patients received non-contrast helical CT scan to evaluate the stone characteristics and peripheral organ disposition. Patients with preoperative positive urine culture received a complete course of culture specific antibiotics treatment. Prophylactic antibiotic was administered to all patients before surgery.

The operative finding, intra- and post-operative complications were recorded. Stones were analyzed using infrared spectroscopy to identify the stone composition. KUB and CT scan was performed to evaluate the stone free status. The success was defined as complete clear or the presence of stone fragment less than 4 mm without any clinical symptoms.

### Surgery procedure

#### Upper urinary tract stone

In lithotomy position, retrograde ureteroscopy for catheterization or ureteral stones was attempted firstly, but all failed due to difficulty in locating the neo-ureteral orifices and in traversing the ureteroenteric anastomosis. The patient was then turned to prone position. The targeted renal calyx was punctured with an 18-gauge needle under sonography guidance. Contrast media was injected into the renal collecting system through the needle sheath and nephrography was administered to check the puncture status, repuncture was arranged if needed. A flexible 0.035-inch flexible guide wire was inserted into renal collecting system under fluoroscopic guidance. The tract was then dilated to 22 Fr using sequential fascial dilators; matched peel-away sheath was inserted.

Kidney stones were fragmented and extracted by ultrasonic lithotripter of Swiss LithoClast Master under nephroscopy. For upper ureteral stone, 8/9.8 Fr semi-rigid ureteroscope (Richard Wolf, Germany) was used to inspect the ureter, stones were fragmented by Ho: YAG laser and stone fragments were extracted by forceps. For distal ureteral stone, flexible ureteroscope (Olympus P5, Japan) was advanced into renal collecting system via the percutaneous tract, and then inserted to the distal ureter following the guide wire. Stones were fragmented by Ho:YAG laser with 200 µm laser fiber. Stone fragments were picked out using 2.2 Fr Nitinol stone basket.

After stone extraction, antegrade urography was performed. In patients without evident ureteral obstruction, a long 5 Fr ureteral catheter was inserted to reservoir with the proximal tip inset into the nephrostomy tube, and removed on postoperative fourth day. If obstruction from uretero-vesical anastomosis was noted, dilation was arranged. A flexible guide wire was inserted into reservoir, the distal tip of guide wire was stretched out form the neo-bladder outflow tract, dilation was performed with long fascial dilator up to 12 Fr, and two double-J stents were indwelled for 2 months. 20 Fr nephrostomy tube was placed at the end of procedure.

#### Reservoir Stone

In 2 patients with reservoir stones after orthotropic urinary diversion, 14 Fr nephroscope was advanced into neo-bladder in a transurethral approach. Stones were fragmented and extracted by ultrasonic lithotripter of Swiss LithoClast Master.

In 2 patients with pouch stones after non-orthotopic urinary diversion, percutaneous pouch lithotripsy was performed. Based on the preoperative CT evaluation results, sonography guided puncture to the pouch was administered, then 0.035-inch flexible guide wire was inserted into pouch. The tract was dilated to 22 Fr using sequential fascial dilators, and 22 Fr peel-away sheath was inserted. Stones were fragmented and extracted by ultrasonic lithotripter. During the operation, fluoroscopy was required to detect the residual stones hide behind the mucosal folds. At the end of procedure, 20 Fr Foley catheter was placed.

In one patient with giant reservoir stones (11.2 cm), open operation was performed, stones were taken out and reservoir conduct was re-established.

## Results

The present study included 18 men and 2 women, with a mean age of 58.3±9.4 years (range 45–72). These cases were 9 patients with ileal conduit (Bricker), 6 patients with colon conduit, 3 patients with ileal orthotopic neobladder (Kock), and 2 patients with Indian Pouch continent diversion. The intervention interval for urinary tract stones to urinary diversion was 27 months (range 15–47 months). The mean stone size was 4.5±3.1 cm (range 1.5–11.2). Kidney stone, ureteral stone and reservoir stones were noted in 8, 3, and 9 patients, respectively. Detailed information for patients' demographics and stone characteristics were list in Table 1.

**Table 1:** Patients' demographics, stone characteristic and treatment results (n = 20).

Index	Value
Age (year)	58.3±9.4 (45–72)
Sex (M:F)	18∶2
Stone size (cm)	4.5±3.1 (1.5–11.2)
Stone location (n, %)	
Kidney	8(40%)
Ureter	3 (15%)
Reservoir	9 (45%)
Preoperative serum creatinine (mg/dl)	1.0±0.4 (0.8–2.0)
Urinary diversion type (n, %)	
Ileal conduit (Bricker)	9 (45%)
Colon conduit	6 (30%)
Ileal orthotopic neobladder(Kock)	3 (15%)
Indian Pouch	2 (10%)
Pathological outcome for previous bladder cancer (n, %)	
Transitional-cell carcinoma (T_1–2_N_0_M_0_)	19 (95%)
Squamous carcinoma (T_2a_N_0_M_0_)	1 (5%)
Intervention received (n, %)	
Percutaneous nephrolithotomy	8 (40%)
Antegrade ureteroscopy	3 (15%)
Percutaneous pouch lithotripsy	6 (30%)
Transurethral neo-bladder lithotripsy	2 (10%)
Open operation	1 (5%)
Operation time (min)	82.0±11.5 (55–120)
Clearance (%)	90% (18/20)
Complications (n, %)	
Fever	2 (10%)
Urine extravasations	1 (5%)
Postoperative serum creatinine (mg/dl)	0.9±0.3 (0.7–1.7)
Stone composition (n, %)	
Calcium oxalate	8 (40%)
Struvite	9 (45%)
Urine acid	1 (5%)
Calcium phosphate	2 (10%)

The mean operation time was 82.0±11.5 min (range 55–120). Eighteen patients were rendered stone free with a clearance of 90%, one case had 5 mm residual stone located in lower pole following percutaneous nephrolithotomy (PCNL), and one patient had 6 mm residual stone in pouch, they received conservative watching treatment. No severe intraoperative complication was noted. Minor postoperative complications were noted in 3 patients (15%, 3/20). Two patients (10%, 2/20) had postoperative fever greater than 38.5°C, one patient with renal calculi received PCNL, and another patient with ureteral stone and uretero-vesical anastomosis obstruction received antegrade ureteroscopy and dilation of obstruction. Both patients received culture specific antibiotics and were cured. One patient (5%, 1/20) suffered urine extravasations from percutaneous tract required delayed extubation, while with good recovery. No transfusion or other severe postoperative complication was noted. Stone composition in this series was listed as follows: calcium oxalate (40%, 8/20), struvite stone (45%, 9/20), calcium phosphate (10%, 2/20), and uric acid (5%, 1/20).

In the 12–48 months follow-up, recurrent bacteriuria were present in 9 patients (45%, 9/20), five patients (25%, 5/20) had persistent hydronephrosis. One patient developed high-grade hydronephrosis resulting from the uretero-vesical anastomosis obstruction, and was treated with incision and dilation of the stricture and indwelling double-J stents. The stone recurrence rate was 20% (4/20). Recurrent kidney stones in 2 patients received conservative observation, and pouch stones in 2 patients were successfully treated with the previous technique. All patients had a stable or improved renal function according to the postoperative SCr level of 0.9±0.3 (range 0.7–1.7)mg/dl, even though there was no statistical significant difference when compared to preoperative SCr level of 1.0±0.4 (range 0.8–2.0) mg/dl, two out of the 4 cases with preoperative renal insufficiency demonstrated normal SCr level, and no patient required dialysis in the follow-up.

## Discussion

Many options have been described for the intervention of urolithiasis in patients after urinary diversion, including PCNL, ureteroscopy, extracorporeal shock wave lithotripsy (SWL), open or laparoscopy operation [10]–[12], [15]–[18]. Comprehensive evaluation and individualized consideration were required, based on the urinary diversion type, stone location, stone burden, available resource and surgeon experience [9]–[10]. Nevertheless, all the studies reported positive results [9]–[12], [16]–[18]. In the present study, patients received minimally invasive surgery got a high stone free rate with minor complications.

The small asymptomatic urolithiasis in patients after urinary diversion always received conservative treatment. SWL was the ideal initial treatment option for patients with small stone burden requiring intervention, given the potential challenges in surgery on account of urinary diversion [17], [21]. The included patients in the present study had a mean stone size of 4.5 cm, therefore, no patient received SWL. In another hand, the fate of stone fragments after SWL was unpredictable; there was great risk of stone reformation in pouch where the stone fragments have little possibility in spontaneous passage.

Regardless of the urinary diversion type, the distortion of lower urinary tract after urinary diversion did not bring great challenge to urologists in performing PCNL in these patients. Exactly, the main difficulties in these cases tend to be in locating the neo-ureteral orifices and retrograde ureteral catheterization [9]. However, sonography can provide excellent guidance in puncture procedure when retrograde urography was not available [19]. We did not experience special difficulties in the PCNL procedure, including in patients needing multiple tracts. Patients with urinary diversion tolerate PCNL well [11], and the success rates ranged from 60% to 86% [9], [23]. Our data with a stone free rate of 87.5% (7/8) for PCNL in patients with urinary diversion was consistent with previous reports [9], [23].

Retrograde ureteroscopy was technically challenging in patients after urinary diversion, as it was hard to get through the neo-ureteral orifice in reservoir. In the study from Delvecchio [15], antegrade advancement of guide wire into neo-bladder, and a subsequent retrograde approach to upper urinary tract stones with flexible ureteroscopy was feasible. However, the time consuming procedure and the need for patients' position changing did not demonstrate significant advantage when compared to the antegrade flexible ureteroscopy. In addition, sometimes, the passage of guide wire through an impacted ureteral stone was impossible. Percutaneous based antegrade ureteroscopy provided an alternative approach for management of ureteral stones. It was possible to inspect the renal pelvis and upper ureter up to L4 through a middle pole percutaneous access with semi-rigid ureteroscope [20]. Furthermore, in the present study, the antegrade flexible ureteroscopy could get to the distal ureter.

The management of reservoir stone differed depending on the urinary diversion type, stone location and burden. A transurethral approach in patients with orthotropic urinary diversion, or a trans-stoma approach in patients with continent diversion, seemed to be ideal. However, excessive torque during the operation might damage the stomal continence mechanism, and also risking in stomal stenosis in a long term [16]. This approach was therefore only recommended in patients with minor stone burden. Percutaneous pouch lithotripsy has been recommended in previous studies [10], [12], [22]. The new generation ultrasonic lithotripter was powerful enough in stone fragmentation and provided stone fragments suction out simultaneously, making the stone extraction procedures much more efficient. However, it was still time consuming for stones with large stone burden. In the other hand, the potential reservoir outlet obstruction required further management rather than an endourological procedure. Open operation for stone extraction and reservoir re-establishment could be performed in some cases, but with great challenge since the tissue scar and adhesion [23]. In the present study, we extracted giant stones (11.2 cm) in one patient and rebuilt the reservoir and outlet tract with open operation, while patients with medium reservoir stone burden were successfully managed with percutaneous pouch lithotripsy, transurethral or trans-stoma approach were only administrated in patients with minor stone burden.

According to the follow up results from the present study, recurrent UTI and hydronephrosis were the most frequently noted issues, underlining the need to concern the reservoir empty capability and uretero-enteric anastomosis obstruction [10]. Management of uretero-enteric anastomosis obstruction, urine culture and subsequent culture specific antibiotics were required to prevent further development of hydronephrosis and related UTI or urolithiasis [10].

The limitation of this retrospective study was the lack of metabolic evaluations, and based on a small cohort of patients from a single center. Further study based on larger series from multiple centers was needed to corroborate our results.

## Conclusions

The percutaneous based procedures, including percutaneous nephrolithotomy, antegrade ureteroscopy with semi-rigid ureteroscope or flexible ureteroscope from percutaneous tract, and percutaneous pouch lithotripsy, provides a direct and safe access to the target stones in patients after urinary diversion, and with high stone free rate and minor complications. The surgical management of urolithiasis in patients after urinary diversion requires comprehensive evaluation and individualized consideration depending upon the urinary diversion type, stone location, stone burden, available resource and surgeon experience.

## References

[1] Daneshmand S, Bartsch G (2011) Improving selection of appropriate urinary diversion following radical cystectomy for bladder cancer. Expert Rev Anticancer Ther 11: 941–948. 21707291 10.1586/era.11.19

[2] Hautmann RE, Hautmann SH, Hautmann O (2011) Complications associated with urinary diversion. Nat Rev Urol 8: 667–677. 22045349 10.1038/nrurol.2011.147

[3] Turk TM, Koleski FC, Albala DM (1999) Incidence of urolithiasis in cystectomy patients after intestinal conduit or continent urinary diversion. World J Urol 17: 305–307. 10552149 10.1007/s003450050151

[4] Terai A, Arai Y, Kawakita M, Okada Y, Yoshida O (1995) Effect of urinary intestinal diversion on urinary risk factors for urolithiasis. J Urol 153: 37–41. 7966785 10.1097/00005392-199501000-00016

[5] Ginsberg D, Huffman JL, Lieskovsky G, Boyd S, Skinner DG (1991) Urinary tract stones: a complication of the Kock pouch continent urinary diversion. J Urol 145: 956–959. 2016808 10.1016/s0022-5347(17)38499-9

[6] Terai A, Ueda T, Kakehi Y, Terachi T, Arai Y, et al (1996) Urinary calculi as a late complication of the Indiana continent urinary diversion: comparison with the Kock pouch procedure. J Urol 155: 66–82. 7490900

[7] Arai Y, Kawakita M, Terachi T, Oishi K, Okada Y, et al (1993) Long-term follow up of the Kock and Indiana pouch procedures. J Urol 150: 51–55. 8510274 10.1016/s0022-5347(17)35394-6

[8] Cohen TD, Streem SB, Lammert G (1996) Long-term incidence and risks for recurrent stones following contemporary management of upper tract calculi in patients with a urinary diversion. J Urol 155: 62–65. 7490899

[9] Cohen TD, Streem SB (1994) Minimally invasive endourologic management of calculi in continent urinary reservoirs. Urology 43: 865–868. 8197652 10.1016/0090-4295(94)90154-6

[10] Okhunov Z, Duty B, Smith AD, Okeke Z (2011) Management of urolithiasis in patients after urinary diversions. BJU Int 108: 330–336. 21615672 10.1111/j.1464-410X.2011.10194.x

[11] Fernandez A, Foell K, Nott L, Fernandez A (2011) Percutaneous nephrolithotripsy in patients with urinary diversions: A case-control comparison of perioperative outcomes. J Endourol 25: 1615–1618. 21823981 10.1089/end.2011.0045

[12] Franzoni DF, Decter RM (1999) Percutaneous vesicolithotomy: an alternative to open bladder surgery in patients with an impassable or surgically ablated urethra. J Urol 162: 777–778. 10458365 10.1097/00005392-199909010-00042

[13] Manohar T, Ganpule A, Desai M (2008) Comparative evaluation of Swiss LithoClast 2 and holmium:YAG laser lithotripsy for impacted upper-ureteral stones. J Endourol 22: 443–436. 18355139 10.1089/end.2007.0288

[14] Faïs PO, Albert T, Gaillet S (2011) Flexible ureteroscopy with laser for upper urinary tract stone. Prog Urol 21: 811–815. 22032607 10.1016/j.purol.2011.10.001

[15] Delvecchio FC, Kuo RL, Iselin CE, Webster GD, Preminger GM (2000) Combined antegrade and retrograde endoscopic approach for management of urinary diversion-associated pathology. J Endourol 14: 251–256. 10795614 10.1089/end.2000.14.251

[16] L'Esperance JO, Sung J, Marguet C, L'Esperance A, Albala DM (2004) The surgical management of stones in patients with urinary diversions. Curr Opin Urol 14: 129–134. 15075843 10.1097/00042307-200403000-00014

[17] El-Assmy A, El-Nahas AR, Mohsen T, Eraky I, El-Kenawy MR, et al (2005) Extracorporeal shock wave lithotripsy of upper urinary tract calculi in patients with cystectomy and urinary diversion. Urology 66: 510–513. 16140067 10.1016/j.urology.2005.04.008

[18] Coleman RL, Mahoney NM, Hatch KD (2002) Laparoscopic management of urolithiasis in a continent urostomy. Gynecol Oncol 84: 473–478. 11855892 10.1006/gyno.2001.6543

[19] Desai M (2009) Ultrasonography-guided punctures-with and without puncture guide. J Endourol 23: 1641–1643. 19715483 10.1089/end.2009.1530

[20] Li X, He Z, Wu K, Li SK, Zeng G (2009) Chinese minimally invasive percutaneous nephrolithotomy: the Guangzhou experience. J Endourol 23: 1693–1697. 19747032 10.1089/end.2009.1537

[21] Cass AS, Lee JY, Aliabadi H (1992) Extracorporeal shock wave lithotripsy and endoscopic management of renal calculi with urinary diversions. J Urol 148: 1123–1125. 1507349 10.1016/s0022-5347(17)36837-4

[22] Roth S, Van Ahlen H, Semjonow A, Von Heyden B, Hertle L (1994) Percutaneous pouch lithotripsy in continent urinary diversions with narrowed Mitrofanoff conduit. Br J Urol 73: 316–318. 8162514 10.1111/j.1464-410x.1994.tb07527.x

[23] Madbouly K (2010) Large orthotopic reservoir stone burden: Role of open surgery. Urol Ann 2: 96–99. 20981195 10.4103/0974-7796.68856 PMC2955233

